# Knowledge, beliefs, and attitudes of spinal manipulation: a cross-sectional survey of Italian physiotherapists

**DOI:** 10.1186/s12998-022-00449-x

**Published:** 2022-09-12

**Authors:** Firas Mourad, Marzia Stella Yousif, Filippo Maselli, Leonardo Pellicciari, Roberto Meroni, James Dunning, Emilio Puentedura, Alan Taylor, Roger Kerry, Nathan Hutting, Hendrikus Antonius Kranenburg

**Affiliations:** 1 Department of Physiotherapy, Exercise and Sports, LUNEX International University of Health, 4671 Differdange, Luxembourg; 2 Luxembourg Health & Sport Sciences Research Institute A.S.B.L., 50, Avenue du Parc des Sports, 4671 Differdange, Luxembourg; 3 Department of Clinical Science and Translation Medicine, Faculty of Medicine and Surgery, University of Rome Tor Vergata, Rome, Italy; 4 Department of Human Neurosciences, Sapienza” University of Rome, Rome, Italy; 5 Sovrintendenza Sanitaria Regionale Puglia INAIL, Bari, Italy; 6 IRCCS Istituto delle Scienze Neurologiche di Bologna, Bologna, Italy; 7 American Academy of Manipulative Therapy Fellowship in Orthopaedic Manual Physical Therapy, Montgomery, AL USA; 8 Montgomery Osteopractic Physiotherapy & Acupuncture Clinic, Montgomery, AL USA; 9 Doctor of Physical Therapy Program, Robbins College of Health and Human Sciences, Baylor University, Waco, TX USA; 10 Faculty of Medicine and Health Sciences, School of Health Sciences, University of Nottingham, Nottingham, UK; 11 Department of Occupation and Health, School of Organisation and Development, HAN University of Applied Sciences, Nijmegen, The Netherlands; 12 Department of Physiotherapy, Hanze University of Applied Sciences, Groningen, the Netherlands

**Keywords:** Manual therapy, Manipulation, Mobilization, Clinical Practice, Hands-off, Hands-on

## Abstract

**Background and Objective:**

High-velocity low-amplitude thrust spinal manipulation (SM) is a recommended and commonly used manual therapy intervention in physiotherapy. Beliefs surrounding the safety and effectiveness of SM have challenged its use, and even advocated for its abandonment. Our study aimed to investigate the knowledge and beliefs surrounding SM by Italian physiotherapists compared with similar practitioners in other countries.

**Methods:**

An online survey with 41 questions was adapted from previous surveys and was distributed via a mailing list of the Italian Physiotherapists Association (March 22–26, 2020). The questionnaire was divided into 4 sections to capture information on participant demographics, utilization, potential barriers, and knowledge about SM. Questions were differentiated between spinal regions. Attitudes towards different spinal regions, attributes associated with beliefs, and the influence of previous educational background were each evaluated.

**Results:**

Of the 7398 registered physiotherapists, 575 (7.8%) completed the survey and were included for analysis. The majority of respondents perceived SM as safe and effective when applied to the thoracic (74.1%) and lumbar (72.2%) spines; whereas, a smaller proportion viewed SM to the upper cervical spine (56.8%) as safe and effective. Respondents reported they were less likely to provide and feel comfortable with upper cervical SM (respectively, 27.5% and 48.5%) compared to the thoracic (respectively, 52.2% and 74.8%) and lumbar spines (respectively, 46.3% and 74.3%). Most physiotherapists (70.4%) agreed they would perform additional screening prior to upper cervical SM compared to other spinal regions. Respondents who were aware of clinical prediction rules were more likely to report being comfortable with SM (OR 2.38–3.69) and to perceive it as safe (OR 1.75–3.12). Finally, physiotherapists without musculoskeletal specialization, especially those with a traditional manual therapy background, were more likely to perform additional screening prior to SM, use SM less frequently, report being less comfortable performing SM, and report upper cervical SM as less safe (*p* < 0.001).

**Discussion:**

The beliefs and attitudes of physiotherapists surrounding the use of SM are significantly different when comparing the upper cervical spine to other spinal regions. An educational background in traditional manual therapy significantly influences beliefs and attitudes. We propose an updated framework on evidence-based SM.

**Supplementary Information:**

The online version contains supplementary material available at 10.1186/s12998-022-00449-x.

## Background

Since the origins of musculoskeletal physiotherapy, high-velocity low-amplitude thrust spinal manipulation (SM) has been widely used by practitioners [1, 2]. SM involves the delivery of a rapid and short impulse to vertebral segments producing joint surface separation that results in intra-articular cavitation commonly accompanied by audible popping sounds [3]. It is suggested that the external force induced by SM, transmitted across the patient’s biological tissues has been found to trigger neurophysiological effects on both the central and the peripheral nervous system [4–7]. In addition, SM has been found to be a cost-effective intervention and to improve patient-reported and performance-based outcome measures [8–12]. Accordingly, SM is an evidence-based intervention which may form part of a management strategy for individuals with a variety of spinal conditions [8–12].

The most recent Clinical Practice Guidelines (CPGs) recommend the inclusion of SM within a multimodal biopsychosocial approach as a first-line treatment for both low back and neck pain [13–15]. Many concerns have been reported regarding serious adverse events (SAEs) following cervical SM [16–18]. Traditionally, pre-manipulative testing has been proposed to help reduce the risk of SAE following cervical SM [19, 20]. Nevertheless, the recent literature suggests that SM may not be the direct cause of SAEs, with a negligible absolute risk of SAE following SM (0.006%) [17, 21–25]. Anxiety regarding safety has been reported as one of the major barriers to the delivery of cervical SM by physiotherapists worldwide [26–28], to such an extent that often even common transitory reactions following manual therapy affecting patient function (e.g., range of motion reduction) are overestimated as adverse responses, especially with regards to the cervical region [29]. These anecdotal increased perception of the actual risk of SM seem to have been further reinforced from sensationalized media coverage [16]. Interestingly, physiotherapists in South Africa (SA) have reported more complications following cervical mobilization rather than cervical SM [30].

Numerous surveys in the United Kingdom (UK), Canada, the United States of America (USA), and the Netherlands have observed that most physiotherapists report high comfort levels when delivering SM to the thoracic and lumbar spines; however, for the cervical spine, increased anxiety on its safety is frequently reported [26–28, 31]. Additionally, high levels of concern regarding the safety of SM have been promoted during musculoskeletal specialization programs (e.g., Orthopaedic Manipulative Physical Therapy [OMPT] and post-graduate programs belonging to the International Federation of Orthopaedic Manipulative Physical Therapists [IFOMPT]) [32].

The curriculum of entry-level physiotherapy programs appears to differ between countries [33, 34]. In the USA, formal training in SM is required by The Commission on Accreditation in Physical Therapy Education (CAPTE) for all entry-level physiotherapy programs. In contrast, SM is not considered an entry-level skill in Italy and is therefore taught in post-graduate programs (e.g., Masters in OMPT, Osteopathy, etc.) or in continuing professional development courses [28, 33]. Many of these educational programs promote differing definitions, technical training, and rationale for the use of SM in clinical practice [1, 35]. Table 1 provides a brief summary of the main differences between these educational programs.

**Table 1:** Differences between the educational programs

Educational program	SM practice
Physiotherapy undergraduate program	A three-years basis bachelor’s degree achieved through a university program. SM is not part of the core-learning outcome. The educational program is mainly focused on orthopedic and neurological rehabilitation and on other area of physiotherapy (e.g., geriatric, pediatric, etc.)
Musculoskeletal specialization	Orthopaedic Manipulative Physical Therapy is a specialized area of physiotherapy for the management of neuro-musculoskeletal conditions. It is achieved through a 2-year post-graduate university program. Many hours are dedicated to the SM practice. SM practice is driven by the available scientific and clinical evidence, and the biopsychosocial framework of each individual patient drive SM practice
Traditional non-thrust manual therapy	Traditional non-thrust manual therapy (e.g., Maitland, Mulligan, Kaltenborn) uses passive and accessory mobilizations of the spine to treat mechanical pain and stiffness on a biomechanical rationale. The qualification is achieved through some modules of few days SM practice involves grades mobilization, with SM as last grade of a progression
Osteopathy post-graduate program	Osteopathy is based on the principle that the body has the ability to heal. In Italy, the qualification is achieved through a non-university post-graduate program with variable duration. Osteopathic SM practice focuses on correcting positional fault (namely, osteopathic lesion) to “facilitate” the normal self-regulatory processes of the body for the treatment of existing conditions and to prevent illness
Continuing professional development course on SM	A heterogenous variety of two-day SM course which includes hands-on practical training and didactic lecture instruction. The main difference between the courses is the background/rationale with which they are delivered and lecturers’ experience, ranging from evidence-based, traditional manual therapy, or osteopathy

SM = Spinal Manipulation

Considereing the inconsistencies in training, knowledge, beliefs and attitudes surrounding the use of SM by physiotherapists, there is a need for an updated evidence-based framework to provide greater standardization in the formal training within entry-level physiotherapy programs across different countries. The primary aims of our study were to: (1) identify the frequency of use of SM in different spinal regions by Italian Physiotherapists; (2) to determine the knowledge and beliefs of Italian physiotherapists on the safety, effectiveness, and perceived barriers surrounding the use of SM within different regions of the spine (i.e., upper cervical, mid-low cervical, thoracic, and lumbar); and (3) to determine the influence of sex, awareness of clinical prediction rules (CPRs), years of practice, practice setting, previous educational background on the use of SM by Italian physiotherapists. A secondary aim was to qualitatively compare the awareness, knowledge, and beliefs on SM by Italian physiotherapists to worldwide physiotherapists. Finally, an updated framework on evidence-based SM was developed.

## Methods

A cross-sectional digital survey was developed using the online platform Survey Monkey (SVMK Inc., San Mateo, USA) addressed to an Italian physiotherapist cohort. The study is reported in line with the Checklist for Reporting Results of Internet Surveys (CHERRIES) [36] and the Strengthening the Reporting of Observational studies in Epidemiology (STROBE) guidelines [37]. This study was approved by the Human Subjects Committee of the Department of Physical Therapy, Occupational Therapy, Rehabilitation and Physical Medicine, Universidad Rey Juan Carlos of Madrid with approval letter URJC-DPTO 37-2020.

### Survey development

After obtaining authors’ permissions, two previous English-written surveys for the USA and the Netherlands were translated and adapted for the Italian setting [26–28]. Given the Italian profession’s historical and socio-legal background, an adjunctive section investigating SM knowledge was implemented. Two PhD physiotherapists with a C1 English language proficiency revised the questionnaire, strengthening the transcultural Italian adaptation and minimizing conceptual ambiguity. The questionnaire was piloted by 5 expert physiotherapists, which provided feedback on clarity of the questions, response logic and completion burden. Construct validity was strengthened using IFOMPT terminology [38] and replication of existing surveys [27, 28, 39]. Content validity was enhanced using multiple sources of evidence-based clinical examination and management strategies [39, 40] including the two previously published surveys [27, 28], and the clinical expert opinion of the authors. The adoption of widely used techniques may also improve the external validity despite the differences between backgrounds and education [27]. To increase the response rate, the survey was designed with 41 close-ended questions.

The questionnaire was divided in to 4 sections: the first investigated the demographics, practice, and education of participants; the second investigated the utilization of SM; and the third investigated potential barriers to the delivery of SM. Sections two and three were sub-divided into lumbar, thoracic, mid-lower cervical (i.e., C3-C7), and upper cervical (i.e., C0-C3) regions. The fourth section investigated participants’ knowledge and perceptions of SM (e.g., indicators to perform, prediction for positive response, popping sound requirement, specificity of the technique, importance within the professional skillset). Because of the heterogeneity of local education on SM, this last section was considered relevant and was additional to previous surveys. All questions were presented in the same order and were mandatory to consider the survey completed (Additional file 1: Appendix 1).

### Setting and recruitment

Initially, the web-link to the survey was distributed via a mailing list of members of the Italian Physiotherapists Association on the March 23, 2020. To maximize the response rate, reminders to participate were redistributed via the same mailing list and published once per week on social media networks (Facebook, Twitter, LinkedIn, and Instagram). The survey was open for one month and the closing date was April 26, 2020. In line with previous internationally published surveys, and for pragmatic purposes, we adopted this methodological approach with the goal to collect the maximum number of answers within a specific period, as most responses occur early after posting [41–46]. Participation was anonymous and voluntary, and informed consent was obtained at the start of the survey. IP addresses were not collected, and the same IP was not allowed to access the survey more than once. No compensation or reimbursement was offered. Participants were able to provide as much information as they desired and were able to stop the survey at any point. According to previous surveys [27, 28], a priori sample size was calculated using the e-survey Dillman’s formula with a 95% confidence level and a 5% of margin of error [47]. At the time of the survey, the number of physiotherapists registered to the association was 7398; therefore, the required sample size for this study was 366 participants.

### Data processing and analysis

The dataset was exported from Survey Monkey to Microsoft Excel 2020 for the purpose of statistical analyses. Analysis of a non-parametric Friedman’s ANOVA with Bonferroni-corrected post-hoc comparisons (through Wilcoxon signed rank test) was used to look for a difference in responses between questions related to: (1) perceived safety and effectiveness of SM by spinal region, (2) additional screening prior to SM by spinal region, (3) utilization of SM, and (4) comfort performing SM by spinal region.

To explore physiotherapists’ attributes associated with their beliefs about SM, ordinal logistic regression was performed for four questions that examined the respondents’ beliefs. Modelled questions were: (1) perceived safety and effectiveness of SM for each spinal regions; (2) whether they regularly provided SM for each spinal region; (3) the level of comfort performing SM for each spinal region; and (4) whether they routinely performed additional screening prior to perform SM for each spinal region.

Finally, in order to investigate the educational programs attended by responders that mostly influenced their utilization of SM in clinical practice, a Kruskal–Wallis test with post-hoc comparisons was run between previous educational programs/background and the questions related to (1) perceived safety and effectiveness of SM by spinal region, (2) additional screening prior to SM by spinal region, (3) utilization of SM, and (4) comfort in performing SM by spinal region. Educational programs were categorized into continuous professional development courses on SM (i.e., any specific SM course with a duration of a few days offered by private, non-academic providers), musculoskeletal specialization (i.e., a master’s degree program following the IFOMPT standard), physiotherapy undergraduate program (a university bachelor’s degree), traditional manual therapy background (e.g., Maitland post-graduate program), osteopathy post-graduate program (i.e., programs provided by private, non-academic providers), and no previous education/training on SM.

All statistical analyses were performed with SPSS 20, (Chicago, IL, USA). The level of significance was set at 0.05 for all comparisons.

## Results

### Responses and respondent characteristics

Five hundred seventy-five (575) Italian physiotherapists completed the survey, accounting for 7.8% of the 7398 target population. Although available for a short period, our sample was in line with previous Italian surveys, and reached the required response rate [48–50]. Four hundred respondents were male (69.6%; 95%CI 6.8–73.3) and 175 were female (30.4%; 95%CI 26.7–32.2). The mean age ± standard deviation (SD) was 32.4 ± 8.31 years. A total of 257 (44.7%; 95%CI 40.6–48.8) physiotherapists held a musculoskeletal specialization. Most respondents worked in a primary care setting (n = 379, 65.9%; 95%CI 62.0–69.8), and 61.6% (n = 354; 95%CI 57.6–65.5) worked in a direct access setting. Forty-eight percent (n = 276, 95%CI 43.9–53.1) had been practicing for 0–5 years and 22.4% (n = 129; 95%CI 19.0–25.8) had practiced no longer than 10 years. Table 2 summarizes the demographic characteristics. The completion of the survey took approximately 10 min.

**Table 2:** Demographic and clinical characteristics of the sample

Variables	N	%	95%CI
*Sex*			
Male	400	69.6	65.8–73.3
Female	175	30.4	26.7–34.2
*Higher degree*			
BSc	292	50.8	46.7–54.9
MSc	258	44.9	40.8–48.9
PhD	25	4.3	2.7–6.0
*Musculoskeletal specialization*			
No	318	55.3	51.2–59.4
Yes	257	44.7	40.6–48.8
*Years of practice*			
0–5	276	48.0	43.9–52.1
6–10	129	22.4	19.0–25.8
11–15	91	15.8	12.8–18.8
16–20	28	4.9	3.1–6.6
20 +	51	8.9	6.5–11.2
*Practice setting*			
Private practice (primary care line)	379	65.9	62.0–69.8
Hospital (secondary care line)	172	29.9	26.2–33.7
Researcher	18	3.1	1.7–4.6
Lecturer	6	1.0	0.2–1.9
*Access regimen*			
Direct access	354	61.6	57.6–65.5
Secondary care referral pathway	221	38.4	34.5–42.4

CI = confidence interval

### Estimate of the most treated spinal regions

Participants were asked to estimate the percentage of patients for each spinal region in their clinical practice. Patients with lumbar complaints were most common (39.9%), followed by the cervical (34.1%), the thoracic spine (14.2%) and the pelvic (11.8%) regions.

### Utilization and awareness of SM clinical prediction rules

Four hundred eighteen (72.7%) participants were aware of CPRs related to SM. Three hundred fifty-six (61.9%) respondents reported being familiar with CPRs about the use of lumbar SM for low back pain; 253 (44.4%) about cervical SM for neck pain; and 238 (41.4%) about thoracic SM for neck pain. Two hundred seventy-four (47.7%) respondents reported using CPRs to help identify those patients who may benefit from SM.

### Perceived safety and effectiveness of SM by spinal region

**Fig. 1:**
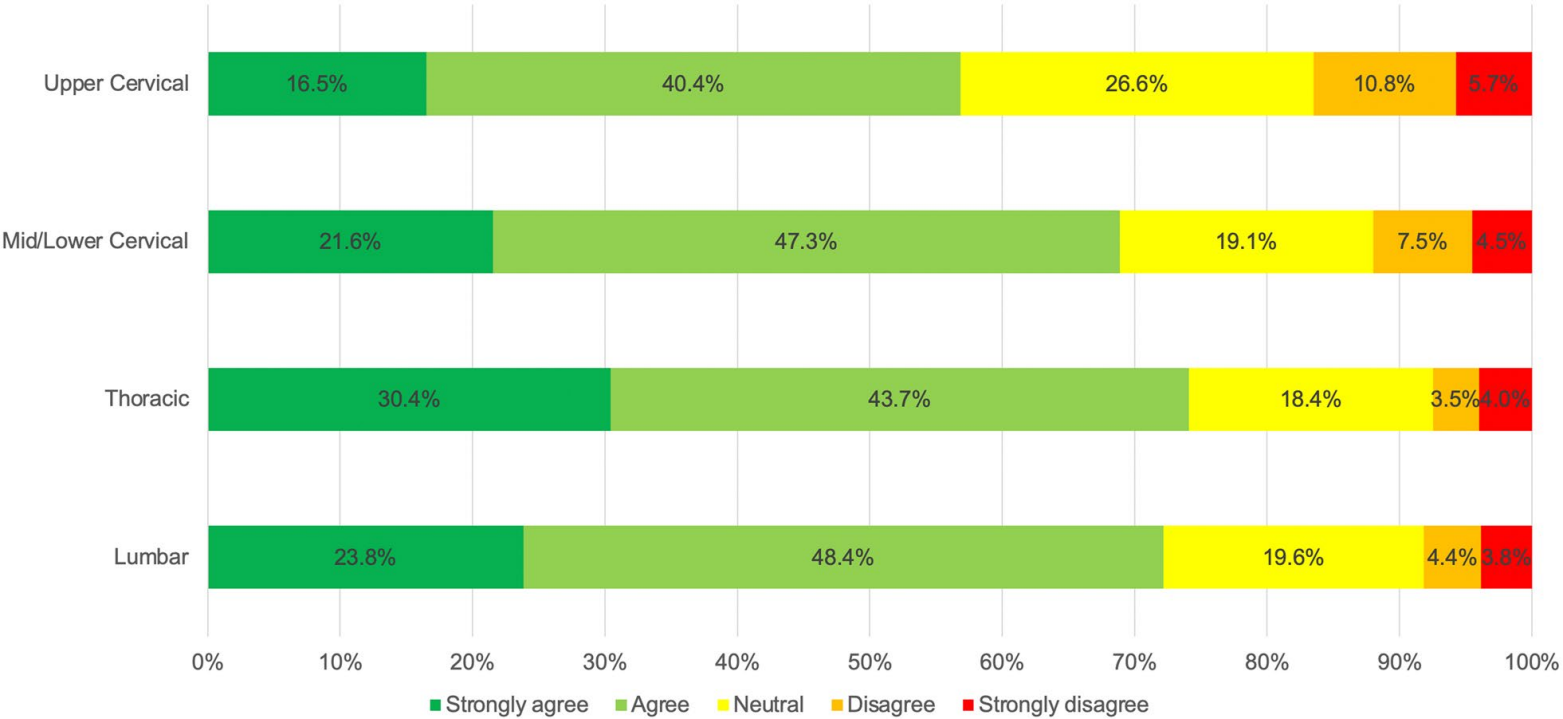
Levels of agreement with the statement ‘SM is safe and effective for patients with XXX complaints’

For levels of agreement with the statement ‘SM is safe and effective for patients with XXX complaints ‘, non-parametric Friedman’s ANOVA revealed a statistically significant difference among spinal regions (χ^2^_df_ = 180.896_3_, *p* < 0.001). Post-hoc Bonferroni-corrected comparisons revealed a significant difference between each of the spinal regions (*p* < 0.003). Most physiotherapists (74.1%) believed that SM was more effective and safer when delivered to the thoracic spine, followed by the lumbar and the cervical spine. Just over half (56.8%) of the respondents reported upper cervical SM as an effective and safe intervention (Fig. 1).

### Additional screening prior to SM by spinal region

**Fig. 2:**
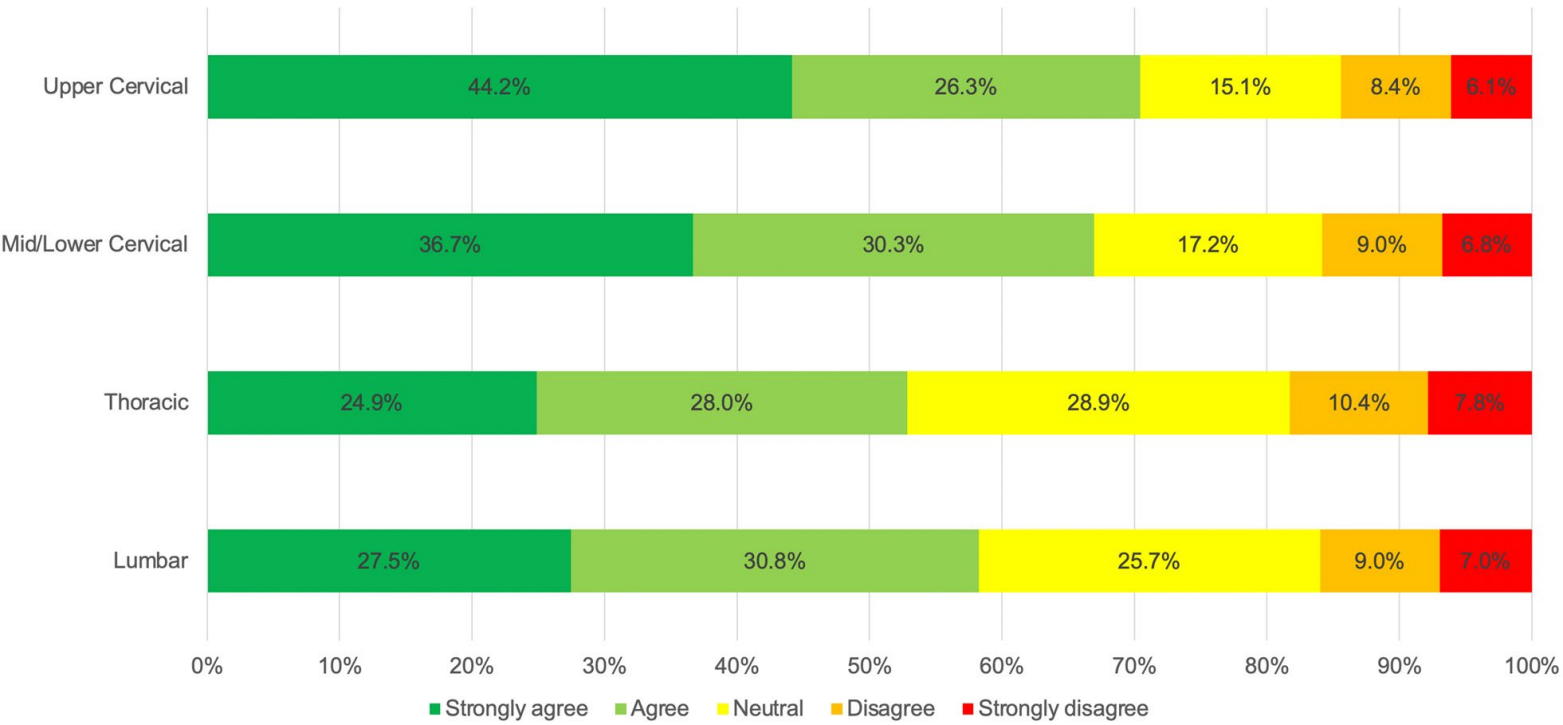
Levels of agreement with the statement ‘Prior to a SM to the XXX spine, I usually perform an additional screening’

For levels of agreement with the statement ‘Prior to a SM to the XXX spine, I usually perform additional screening’ a statistically significant difference was found among the spinal regions (χ^2^_df_ = 252.932_3_, *p* < 0.001). Post-hoc comparisons revealed a significant difference between each of the spinal regions (*p* < 0.001). Most respondents reported they would perform additional screening prior to the use of upper cervical SM more often than to the mid-lower cervical spine; to the cervical spine more often than the lumbar and thoracic spine; and to the lumbar spine more often than for the thoracic spine. Notably, 70.4% of respondents reported performing additional screening prior to the use of upper cervical SM; whereas, 52.9% of respondents reported performing additional screening prior to the use of thoracic SM (Fig. 2).

### Utilization of spinal manipulation

**Fig. 3:**
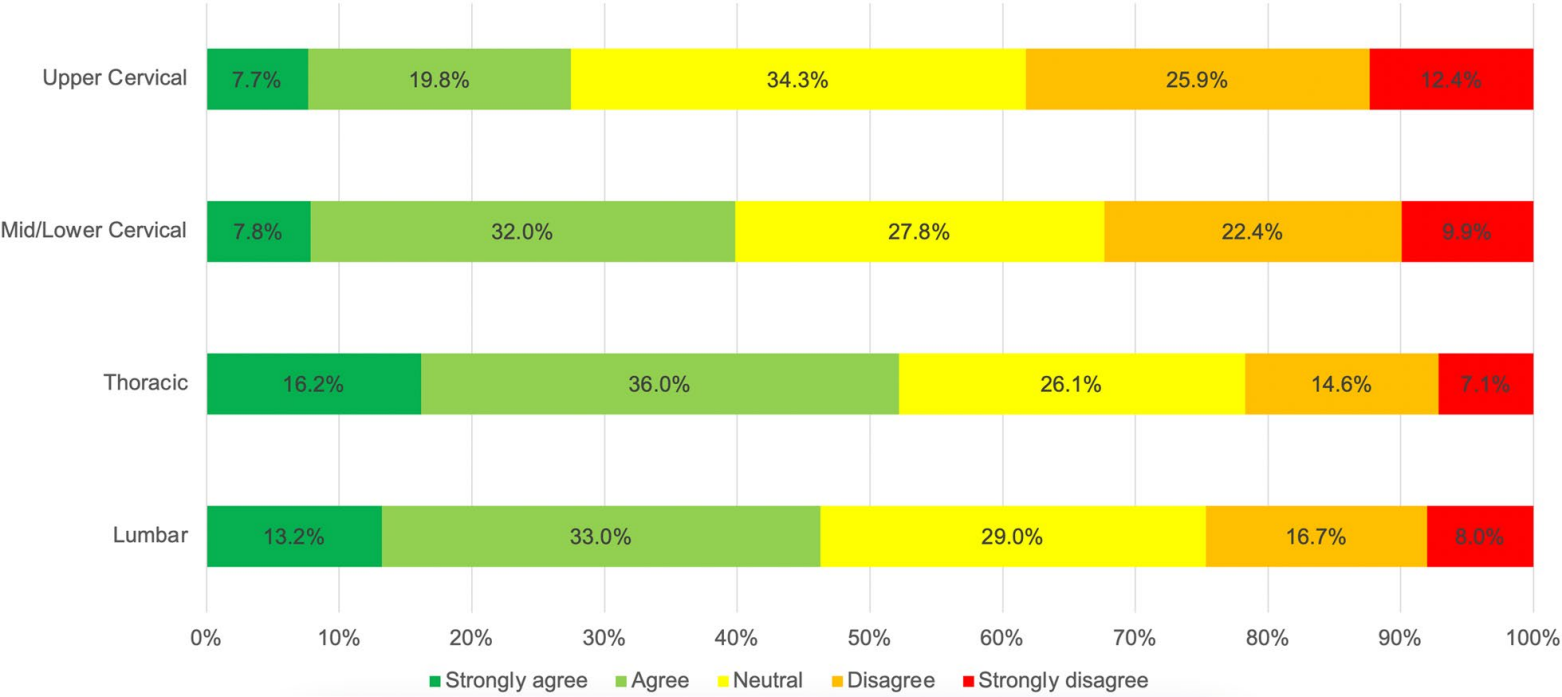
Levels of agreement with the statement ‘I regularly provide SM to the XXX spine when patients require it’

For levels of agreement with the statement ‘I regularly provide SM to the XXX spine when patients require it’ non-parametric Friedman’s ANOVA revealed a statistically significant difference among the spinal regions (χ^2^_df_ = 252.932_3_, *p* < 0.001). Post-hoc comparisons revealed a significant difference between each of the spinal regions (*p* < 0.001). Respondents reported that they regularly delivered SM to the thoracic spine more often than to the lumbar spine, more often to the lumbar spine than to the cervical spine, and more often to the mid-lower cervical spine than to the upper cervical spine. Respondents reported utilizing SM to the thoracic spine most often (52.2%) and least frequently to the upper cervical spine (27.5%) (Fig. 3).

### Comfort performing SM by spinal region

**Fig. 4:**
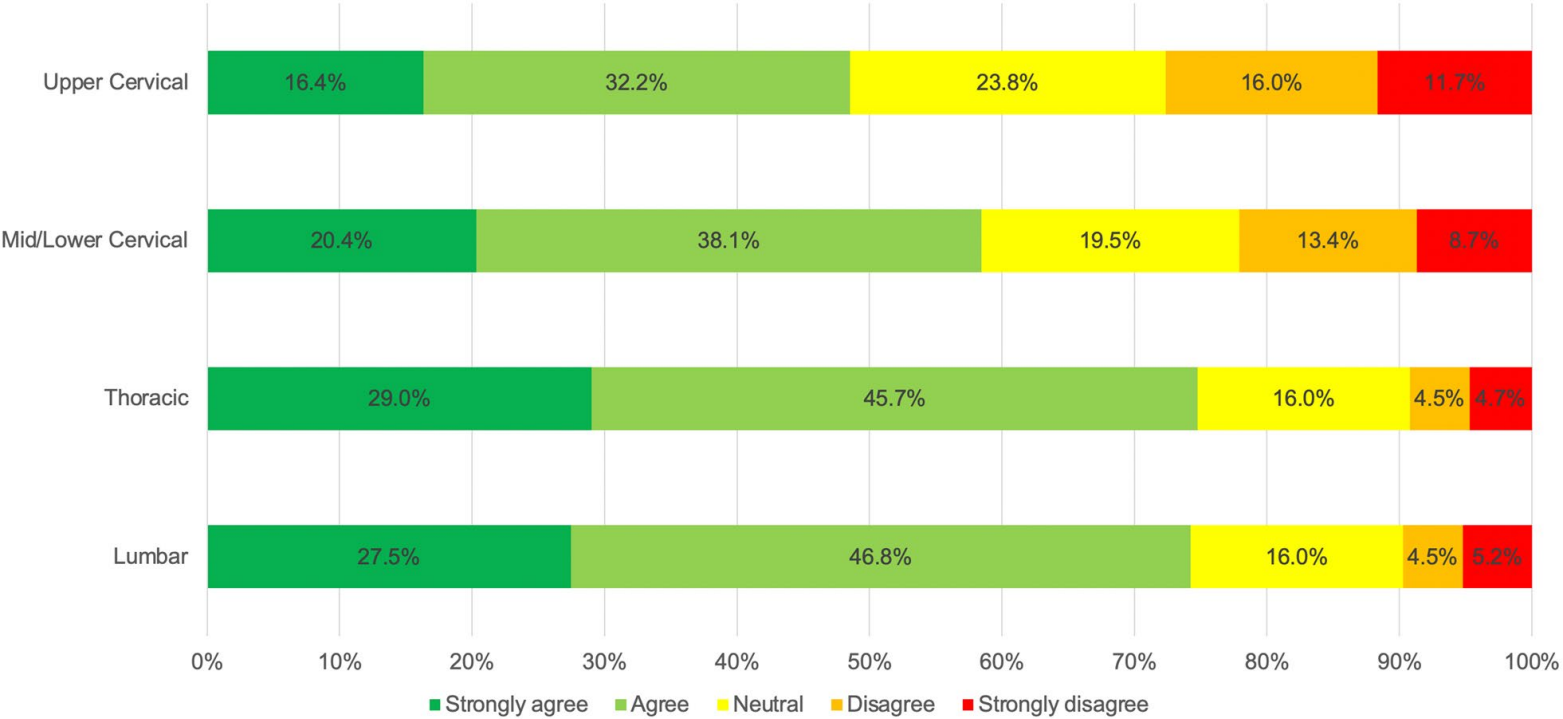
Levels of agreement with the statement ‘I am comfortable performing SM to the XXX spine when patients require it’

For levels of agreement with the statement ‘I am comfortable performing SM to the XXX spine when patients require it’ a statistically significant difference was found among the spinal regions (χ^2^_df_ = 424.558_3_, *p* < 0.001). Post-hoc comparisons revealed a significant difference between each of the spinal regions (*p* < 0.001), except between the lumbar and thoracic spine (p = 0.136). Notably, 48.5% of participants agreed that they were comfortable delivering upper cervical SM, while 74.8% and 74.3%, respectively, reported being comfortable utilizing thoracic and lumbar SM (Fig. 4).

### Barriers to SM delivery

**Fig. 5:**
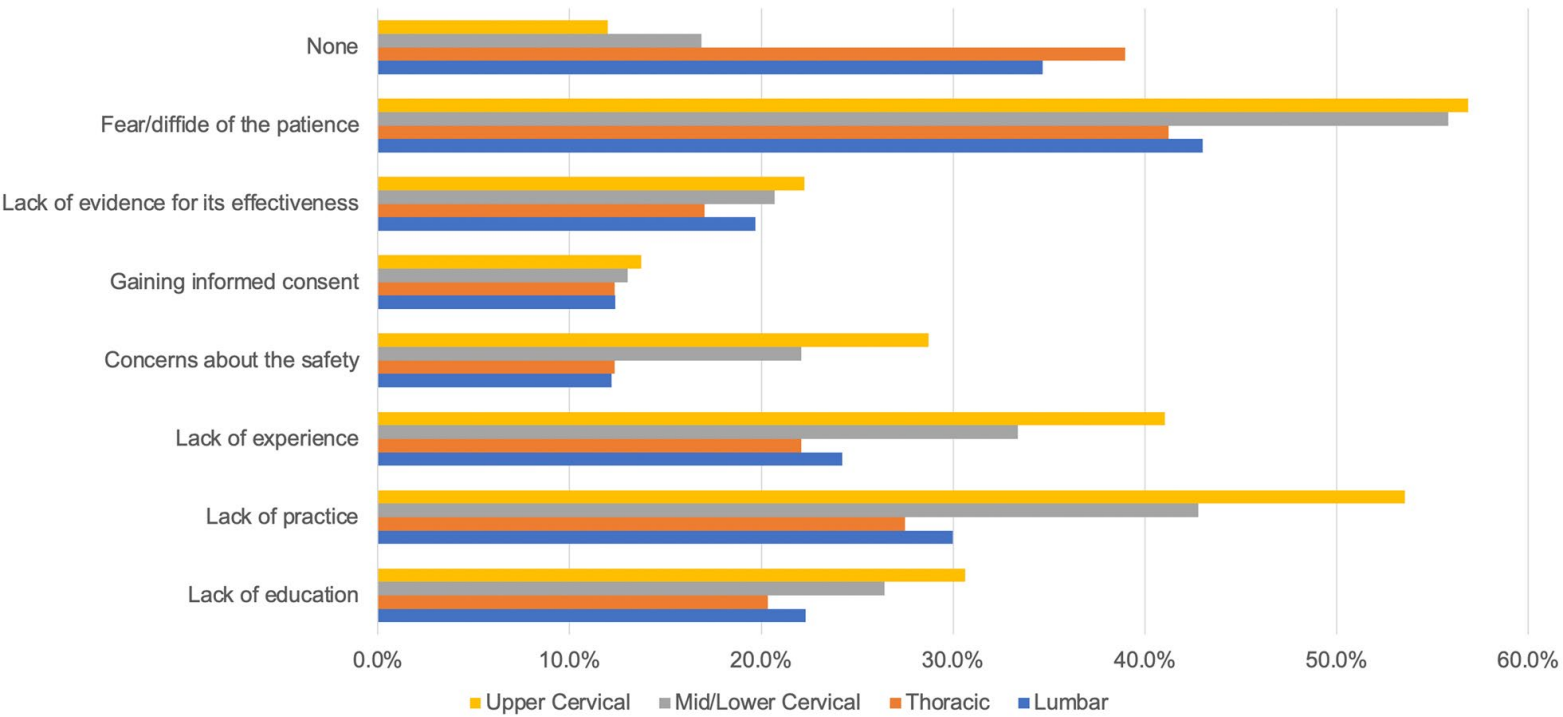
Barriers to the use of SM by Italian physiotherapists

Patients’ fear (n = 327, 56.9%; n = 321, 55.8%), lack of clinicians’ formal training (n = 308, 53.6%; n = 246, 42.8%) and lack of clinical experience (n = 236, 41%; n = 237, 41.2%) were the most frequently reported barriers, respectively, for using SM to the upper cervical and mid-lower cervical spines. Patients’ fear (n = 237, 41.2%; n = 247, 43%) and lack of practice/training (n = 158, 27.5%; n = 172, 29.9%) were the most frequently reported barriers, respectively, for the use of thoracic and lumbar SM in clinical practice. Only 12% of the respondents reported no barriers in the delivery of SM to the upper cervical spine, while 39.0% and 34.6%, respectively, reported no barriers to the utilization of SM to the thoracic and lumbar spines (Fig. 5).

### Modelling

For all four modelled questions, individual models were generated for each of the spinal regions, such that a total of sixteen models were created. Sex, awareness of CPRs, years of practice, musculoskeletal specialization, and practice setting were used in each ordinal logistic regression to adjust all models.

Depending on the spinal region, the odds of physiotherapists being aware of CPRs reporting SM as safe and effective were 1.75–3.12 times greater compared to those unaware of the CPRs. The odds of physiotherapists with 6–10 years of clinical practice reporting SM as safe and effective were 1.75–2.15 greater compared to those practicing for 0–5 years. The odds of physiotherapists without musculoskeletal specialization reporting upper cervical SM as safe and effective were 1.79 smaller compared to those possessing a musculoskeletal specialization (Table 3).

**Table 3:** Ordinal logistic regression model results for the question ‘SM is safe and effective for patients with XXX complaints’

	Upper cervical OR (95% CI)	Mid/lower cervical OR (95% CI)	Thoracic OR (95% CI)	Lumbar OR (95% CI)
*Sex*				
Male	**2.11** (1.44–3.09)	**2.03**(1.37–3.01)	1.22 (0.81–1.84)	1.48 (0.98–2.24)
Female	Ref	Ref	Ref	Ref
*CPR aware*				
Yes	**1.75** (1.18–2.61)	**1.96** (1.31–2.95)	**2.12** (1.39–3.22)	**3.12** (2.07–4.74)
No	Ref	Ref	Ref	Ref
*Years of practice*				
0–5	Ref	Ref	Ref	Ref
6–10	**1.92** (1.31–2.82)	**2.15** (1.48-.3.21)	**2.01** (1.33–3.02)	**1.71** (1.14–2.57)
*Musculoskeletal specialization*				
Yes	Ref	Ref	Ref	Ref
No	**1.79** (1.25–2.58)	1.26 (0.86–1.86)	0.96 (0.65–1.44)	**0.85** (0.57–1.26)
*Access regimen*				
Direct access	**1.76** (1.22–2.54)	**1.51** (1.02–2.21)	1.37 (0.92–2.05)	1.12 (0.75–1.66)
Secondary care referral pathway	Ref	Ref	Ref	Ref

Bold OR’s indicate significant results; CPR = clinical prediction rule; OR = odds ratio; CI = confidence interval

Depending on the spinal region, the odds of physiotherapists without musculoskeletal specialization to routinely perform additional screening prior to SM were 2.55 and 2.18 times greater for the upper and mid/lower cervical spine, respectively, compared to those with musculoskeletal specialization (Table 4).

**Table 4:** Ordinal logistic regression model results for the question ‘Prior to a SM to the XXX spine, I usually perform an additional screening’

	Upper cervical OR (95% CI)	Mid/lower cervical OR (95% CI)	Thoracic OR (95% CI)	Lumbar OR (95% CI)
*Sex*				
Male	0.88 (0.58–1.34)	0.76 (0.51–1.15)	0.89 (0.62–1.31)	0.72 (0.48–1.05)
Female	Ref	Ref	Ref	Ref
*CPR aware*				
Yes	**2.55** (1.65–3.91)	**2.18**(1.43–3.34)	1.41 (0.95–2.08)	**1.67** (1.12–2.49)
No	Ref	Ref	Ref	Ref
*Years of practice*				
0–5	1.43 (0.95–2.14)	**1.56** (1.05–2.32)	1.28(0.88–1.87)	1.17 (0.81–1.72)
6–10	Ref	Ref	Ref	Ref
*Musculoskeletal specialization*				
Yes	Ref	Ref	Ref	Ref
No	**0.33** (0.22–0.48)	**0.29** (0.20–0.43)	**0.39** (0.28–0.56)	**0.36** (0.25–0.52)
*Access regimen*				
Direct access	1.01(0.67–1.49)	0.88 (0.61–1.31)	0.76 (0.53–1.09)	0.95 (0.66–1.36)
Secondary care referral pathway	Ref	Ref	Ref	Ref

Bold OR’s indicate significant results; CPR = clinical prediction rule; OR = odds ratio; CI = confidence interval

Depending on the spinal region, the odds of males regularly performing SM were 1.65–2.49 times greater compared to females. The odds of physiotherapists who are aware of CPRs regularly performing SM were 2.01–2.45 times greater compared to physiotherapists unaware of the CPRs. The odds of physiotherapists without musculoskeletal specialization regularly performing SM were 1.49–2.01 smaller compared to those possessing a musculoskeletal specialization (Table 5).

**Table 5:** Ordinal logistic regression model results for the question ‘I regularly provide SM to the XXX spine when patients require it’

	Upper cervical OR (95% CI)	Mid/lower cervical OR (95% CI)	Thoracic OR (95% CI)	Lumbar OR (95% CI)
*Sex*				
Male	**2.49** (1.56–3.99)	**2.01** (1.35–2.98)	**1.71** (1.17–2.49)	**1.65** (1.13–2.42)
Female	Ref	Ref	Ref	Ref
*CPR aware*				
Yes	1.51 (0.94–2.42)	**2.01** (1.32–3.05)	**2.45** (1.65–3.65)	**2.38** (1.59–3.57)
No	Ref	Ref	Ref	Ref
*Years of practice*				
0–5	0.75 (0.49–1.13)	0.93 (0.64–1.37)	1.05 (0.72–1.53)	0.96 (0.66–1.41)
6–10	Ref	Ref	Ref	Ref
*Musculoskeletal specialization*				
Yes	Ref	Ref	Ref	Ref
No	**2.01** (1.36–2.97)	**1.49** (1.04–2.13)	1.23 (.87–1.75)	1.27 (0.90–1.80)
*Access regimen*				
Direct access	1.48 (0.98–2.23)	1.37 (0.95–1.98)	1.34 (0.94–1.91)	1.08 (0.76–1.55)
Secondary care referral pathway	Ref	Ref	Ref	Ref

Bold OR’s indicate significant results; CPR = clinical prediction rule; OR = odds ratio; CI = confidence interval

Depending on the spinal region, the odds of male physiotherapists being more comfortable with performing SM were 2.62–3.42 times greater compared with females. The odds of physiotherapists being more comfortable with performing SM who are aware of CPRs was 2.38–3.69 times greater than that of physiotherapists unaware of the CPRs. The odds of physiotherapists without musculoskeletal specialization comfortable with upper cervical SM was 1.92 times smaller compared to those possessing a musculoskeletal specialization. Finally, the odds of those working in a direct access setting being comfortable with performing SM was 1.49–1.75 times greater compared to physiotherapists working in a secondary care referral pathway (Table 6).

**Table 6:** Ordinal logistic regression model results for the question ‘I am comfortable performing SM to the XXX spine when patients require it’

	Upper cervical OR (95% CI)	Mid/lower cervical OR (95% CI)	Thoracic OR (95% CI)	Lumbar OR (95% CI)
*Sex*				
Male	**3.42** (2.27–5.13)	**3.24** (2.19–4.79)	**2.78** (1.82–4.26)	**2.62** (1.73–3.99)
Female	Ref	Ref	Ref	Ref
*CPR aware*				
Yes	**2.38** (1.56–3.62)	**2.61** (1.74–3.91)	**3.69** (2.39–5.72)	**3.13** (2.03–4.81)
No	Ref	Ref	Ref	Ref
*Years of practice*				
0–5	**1.52** (1.02–2.26)	**1.57** (1.05–2.33)	**2.15** (1.38–3.33)	**2.17** (1.42–3.34)
6–10	Ref	Ref	Ref	Ref
*Musculoskeletal specialization*				
Yes	Ref	Ref	Ref	Ref
No	**1.92** (1.33–2.78)	**1.61** (1.11–2.34)	1.04 (0.68–1.61)	.96 (0.63–1.46)
*Access regimen*				
Direct access	**1.75** (1.21–2.55)	**1.49** (1.03–2.17)	**1.69** (1.11–2.58)	**1.57** (1.04–2.38)
Secondary care referral pathway	Ref	Ref	Ref	Ref

Bold OR’s indicate significant results; CPR = clinical prediction rule; OR = odds ratio; CI = confidence interval

### Knowledge and beliefs of the SM by Italian physiotherapists

Reduced mobility (n = 374, 65%), patient’s expectation (n = 337, 58.6%), pain (n = 331, 57.6%), CPRs (n = 315, 54.8%) and physical examination (n = 245, 42.6%) were the main reported factors leading to the decision to deliver SM. SM was considered to have been successfully performed when: “symptoms improved” (n = 420, 73%), “when mobility (general or accessory) improved” (n = 371, 64.5%), “when multiple popping sounds were elicited” (n = 285, 49.6%), and “when a single popping sound was heard” (n = 170, 29.6%). “No popping sound” (n = 54, 9.4%) and “positional-fault repositioning” (n = 54, 9.4%) were the least reported responses.

Most physiotherapists (n = 493; 85.7%) considered the popping sound as a physical phenomenon associated with intra-articular gas release (i.e., cavitation). Three hundred seventy-six participants (65.4%) did not consider the popping sound as necessary. Four hundred fifty (78.3%) reported being aware of the limits of specifically targeting a spinal segment and 498 (86.6%) of eliciting the popping sound on a specific spinal segment. Interestingly, for most of the respondents (n = 458; 79.7%), non-thrust mobilization was considered as the first-choice manual therapy intervention (i.e., to be used instead of or prior to SM). Generally, Italian physiotherapists considered SM not to be an important part of their core skill set (mean = 46.5 on a 100-Likert scale).

### Influence of previous educational background

Table 7 shows the mean (± SD) responses in the questions related to: (1) perceived safety and efficacy of SM by spinal region, (2) additional screening before SM by spinal region, (3) use of SM, and (4) comfort in performing SM by spinal region among previous educational programs/backgrounds influencing SM practice. The Kruskal–Wallis test showed significant differences in all the answers between the groups (*p* < 0.001). In summary, post-hoc comparisons revealed differences between respondents with a musculoskeletal specialization and those who attended traditional manual therapy post-graduate programs (i.e., Maitland). Also, differences were found among respondents who attended a continuing professional development course on SM and those with a postgraduate traditional manual therapy program (i.e., Maitland). Differences were significant for questions on perceived safety and efficacy, use of SM and comfort in performing SM, but not in the question related to additional screening before SM (Additional file 2: Appendix 2). Specifically, those with a musculoskeletal specialization reported more frequently that SM was safer and more effective, that SM was performed more regularly, and felt more comfortable with performing SM compared to respondents with a traditional manual therapy background (i.e., Maitland) (Table 6). Notably, the latter most often reported performing additional screening prior to utilizing SM compared to those with musculoskeletal specialization. Finally, the post-hoc comparisons did not reveal any significant difference in all questions among responders who gained a musculoskeletal specialization and responders who attended continuing professional development courses on SM (Additional file 2: Appendix 2).

**Table 7:** Response on safety and effectiveness of SM by spinal region, additional screening prior to SM by spinal region, utilization of SM, and comfort performing SM by spinal region for each educational programs influencing the clinical practice

Question	Spinal region	MSK	CPD	PT	TRAD	OSTEO	NONE
SM is safe and effective for patients with XXX spine complaints	Upper cervical (C0-3)	2.11 ± 0.85	2.25 ± 1.01	2.78 ± 1.06	3.14 ± 1.07	2.47 ± 1.06	2.88 ± 1.16
	Cervical (C3-7)	1.94 ± 0.78	2.06 ± 0.97	2.47 ± 1.01	2.79 ± 1.13	2.4 ± 1.11	2.47 ± 1.05
	Thoracic	1.77 ± 0.77	1.92 ± 0.98	2.18 ± 0.91	2.41 ± 1.09	2.27 ± 1.04	2.44 ± 1.18
	Lumbar	1.88 ± 0.81	2.06 ± 0.93	2.24 ± 0.81	2.69 ± 1.07	2.16 ± 0.94	2.26 ± 1.09
Prior to a SM to the XXX spine, I usually perform an additional screening	Upper cervical (C0-3)	2.27 ± 1.24	2.13 ± 1.22	2.14 ± 1.08	1.92 ± 1.26	1.64 ± 1.08	1.88 ± 1.14
	Cervical (C3-7)	2.37 ± 1.22	2.31 ± 1.24	2.16 ± 1.05	2.05 ± 1.25	1.95 ± 1.17	1.88 ± 1.20
	Thoracic	2.61 ± 1.16	2.64 ± 1.16	2.35 ± 1.11	2.33 ± 1.22	2.38 ± 1.24	2.19 ± 1.31
	Lumbar	2.56 ± 1.17	2.51 ± 1.15	2.29 ± 1.08	2.26 ± 1.22	2.19 ± 1.16	1.86 ± 1.15
I regularly perform SM to the XXX spine when patients require it	Upper cervical (C0-3)	2.97 ± 1.03	2.97 ± 1.11	3.29 ± 0.94	3.70 ± 1.03	2.84 ± 1.18	3.49 ± 1.20
	Cervical (C3-7)	2.68 ± 1.05	2.79 ± 1.10	3.10 ± 1.01	3.38 ± 1.12	2.79 ± 1.13	3.58 ± 1.07
	Thoracic	2.27 ± 1.00	2.47 ± 1.09	2.84 ± 1.03	3.10 ± 1.19	2.49 ± 1.12	3.14 ± 1.19
	Lumbar	2.45 ± 1.02	2.58 ± 1.14	3.00 ± 1.02	3.21 ± 1.11	2.63 ± 1.10	3.07 ± 1.30
I am comfortable performing SM to the XXX spine when patients require it	Upper cervical (C0-3)	2.43 ± 1.15	2.45 ± 1.11	3.18 ± 1.11	3.42 ± 1.24	2.41 ± 1.20	3.33 ± 1.19
	Cervical (C3-7)	2.12 ± 1.06	2.23 ± 1.01	2.94 ± 1.12	3.25 ± 1.24	2.29 ± 1.20	3.16 ± 1.17
	Thoracic	1.81 ± 0.79	1.81 ± 0.81	2.39 ± 1.00	2.63 ± 1.23	1.92 ± 0.88	2.84 ± 1.23
	Lumbar	1.82 ± 0.83	1.92 ± 0.82	2.39 ± 1.00	2.70 ± 1.17	1.97 ± 1.04	2.65 ± 1.23

SM = high-velocity low-amplitude spinal thrust manipulation; MSK = musculoskeletal specialization; PT = physiotherapy undergraduate program; CPD = continuing professional development courses on SM; OSTEO = Osteopathy post-graduate program; TRAD = traditional manual therapy post-graduate programs (i.e., Maitland); NONE = never been trained. Data are presented as mean ± standard deviation. Lower scores correspond to a greater degree of agreement, while higher scores correspond to a lesser degree of agreement

## Discussion

### Key findings

To the best of our knowledge this study is the first to investigate Italian physiotherapists’ knowledge, attitudes, and beliefs about SM, and the influence of prior educational background on SM practice. Our results provide validation of previous surveys conducted in the USA and the Netherlands; more specifically, this survey confirms that physiotherapists’ beliefs on the safety and effectiveness of SM strongly differ between the cervical spine and other spinal regions [27, 28]. Similar to British, Canadian, American, and Dutch physiotherapists, Italian physiotherapists reported that they regularly use SM in the management of their patients for a variety of musculoskeletal conditions; furthermore, Italian physiotherapists reported being comfortable in the delivery of SM to the thoracic spine, but less so for the cervical spine [26–28, 31]. Notably, the utilization, the comfort, and perceived safety for upper cervical SM by Italian physiotherapists differs considerably from other spinal regions with several barriers being identified [27]. Respondents with a background in traditional non-thrust manual therapy (i.e., Maitland) reported using SM significantly less often, perceived SM as less safe and less effective, and reported less comfortable with performing SM; furthermore, they reported more frequently performing additional screening prior to the use of SM.

### Beliefs about safety and the influence of educational programs

Similar to previous surveys, we observed that most physiotherapists generally agreed that SM is safe and effective in all spinal regions [26–28, 31, 51]. Although reviews with less methodological flaws have demonstrated that SM is safe and a causal link between SM and SAEs is still unproven [18], physiotherapists from SA, UK, Canada, the USA, the Netherlands, and Italy reported cervical SM—especially upper cervical SM–the least safe and effective [26–28, 31, 51]. Although extensive literature on the safety of SM relies mainly on communicated opinions and on poor-quality non-systematic reviews [22, 34], one reason could be that most of the studies investigating SAEs following SM were focused on the cervical spine [22]. Furthermore, these beliefs appear to be mainly based on anecdotal reports rather than high-quality clinical studies, and concerns about the safety of cervical SM are still consistently propagated within post-graduate programs and academic settings [52–55].

Adams and Sim (1998) were the first to observe that educational programs have a direct influence on practice—i.e., many UK physiotherapists have a background in the Maitland paradigm (i.e., a traditional non-thrust manual therapy approach); hence, not surprisingly, they emerged as being less frequent users of SM [26]. Similarly, in our survey, Italian physiotherapists with a background in a traditional non-thrust manual therapy paradigm (i.e., Maitland) tended to utilize SM less frequently for all spinal regions; furthermore, they reported SM as less safe, less effective, and being less comfortable with the technical delivery of SM techniques in clinical practice. “Lack of practice/training” have been consistently reported as one of the major barriers to performing SM [26–28, 31]; that is, possessing a musculoskeletal specialization, having attended a continuing professional development course on SM, or an osteopathy program are significantly associated with higher levels of reported comfort with performing SM when compared to not having previous education/training on SM and having a background in a traditional non-thrust manual therapy approach. Interestingly, no difference was found between the latter; moreover, one reason for this could be that SM receives less emphasis and less formal training when it comprises just one small aspect of a traditional non-thrust manual therapy education program [35]. Furthermore, in our study, physiotherapists with backgrounds in traditional non-thrust manual therapy paradigms reported the most frequent use of pre-manipulative testing for the upper cervical spine. Therefore, when physiotherapists with a background in traditional non-thrust manual therapy consider the use of SM, especially to the upper cervical spine, it appears their beliefs on the risks outweigh the perceived effectiveness.

For the reasons above, widespread confirmation bias may have influenced clinicians to consider non-thrust mobilization as a safer approach and led researchers to investigate non-thrust mobilization as an alternative intervention to SM [1, 31, 56–60]. Moreover, perhaps due to the perceived risk that is mainly based on anecdotal reports, many researchers have avoided investigating the effectiveness of SM to the cervical region, and have instead focused on investigating alternative approaches such as thoracic manipulation for the treatment of neck pain [61, 62]. In addition, all of the above may be some of the reasons why these two very different treatment techniques progressively became considered by some to be synonymous, interchangeable, or a progression of the same approach (namely, mobilization/manipulation) [1, 31, 56–59], as observed by the preference to use mobilization as the first-choice treatment by Italian physiotherapists.

Another example of the influence of educational background concerns the phenomena of audible ‘popping’ sounds during SM. Although the etiology of the popping sounds is still under debate, the collapse of gas bubbles within the zygapophyseal (i.e., facet) joints (i.e., the “cavitation” phenomenon) has traditionally been accepted as the main mechanism [63, 64], as well as for most Italian physiotherapists. Nevertheless, a more recent study provided tribonucleation as an alternative mechanism to the popping sound [65]—that is, the use of cine MRI appeared to confirm that the audible popping sounds occurred at cavity inception, and no bubble/cavity collapse was ever visualized. Furthermore, several recent studies reported a mean of 3–4 audible popping sounds following thrust spinal manipulation to the upper cervical spine, cervicothoracic junction, and lumbosacral spine [66–68]. Therefore, the cavitation hypothesis alone appears unable to explain the multiple audible popping sounds, and the different frequencies and waveforms associated with these sounds, following SM [66–68].

The popping sound is still a controversial topic for both the effectiveness (i.e., by influencing clinical outcomes) and the definition of SM. Notably, IFOMPT included the popping sound in its definition of SM [3]; furthermore, Evans and Lucas [69] concluded that the popping sound is one of the five necessary criterion for a valid definition of SM. Nevertheless, in the current survey, most Italian physiotherapists did not consider the popping sound as an important indicator for the successful technical delivery of SM. The notion that the popping sound is not related to clinical outcomes originates from a few studies [70–73] and traditional approaches [35]. However, most practitioners anecdotally believe that the popping sounds are an indicator of the successful delivery of SM, explaining why researchers often repeat SM when the popping sound is not elicited on the first attempt [10, 66, 74–82]. In addition, some studies have observed preliminary evidence that suggests a greater hypoalgesic effect (i.e., perhaps associated with proinflammatory cytokine secretion, temporal sensory summation, and/or supraspinal mechanisms) in subjects that experienced audible popping [73, 83, 84]. Although no firm conclusions can be drawn about the clinical relevance, when the popping sound was a requirement in the methods, SM was found more effective in reducing short-term pain and disability than non-thrust mobilization (i.e., no audible popping sound) [74, 82]. Interestingly, patients themselves appear to expect popping sounds to accompany SM [85, 86]. Therefore, the assertion that the popping sound is not required for a successful SM is not supported by the three-pillars of evidence-based practice [87].

### Clinical prediction rules and additional screening prior to SM

Prescriptive CPRs has been designed to help guide clinical decision making to provide SM only to those patients that are likely to benefit from such treatments and to attempt to reduce the presumed risks of SM [19]. Even though multiple systematic reviews have raised concerns about the value of these prediction rules and the validation of these tools [19, 88], similarly to Puentedura et al., we found that being aware of spine CPRs impacted the beliefs on safety, the perception of effectiveness, and the utilization of SM [28]. Notably, the fact remains that none of the prescriptive CPRs in physiotherapy are recommended for application in clinical practice as validation and impact analysis studies are still lacking [89].

With the attempt to identify those patients at increased risk of having an SAE following SM, and according to researchers and academics that still recommend its use, [20] a significant proportion of our survey participants agreed with using pre-manipulative testing for the upper cervical spine. However, the validity of pre-manipulative testing for screening purposes has been questioned due to the low sensitivity and low specificity of the test procedures themselves [90–93]. Thus, because of a lack of construct validity, questionable safety, and an inability to predict SAEs, IFOMPT and many researchers have recommended pre-manipulative testing be abandoned [25, 40, 50, 68, 94, 95].

These widely established beliefs seem to outweigh the impact of current literature on physiotherapists’ practice; hence, de-implementation of the clinical use of these pre-manipulative tests appears to be challenging. Undergraduate programs have been observed not being able to translate updated literature from academic training to clinical settings [96]. However, we observed that possessing a musculoskeletal specialization decreases the odds of using CPRs and additional pre-manipulative testing prior to SM, positively impacting the implementation of current literature into practice.

### SM utilization, comfort, and barriers

Half of the Italian physiotherapists reported regularly utilizing SM, but only one quarter would regularly provide upper cervical SM. Notably, this utilization rate is similar to Canadian and the USA physiotherapists [28, 31]. However, these results are much lower than the rates reported by physiotherapists in the Netherlands and UK [26, 27]. This may be explained by the fact that Kranenburg et al. [27] and Adams and Sim [26] limited their survey to musculoskeletal physiotherapists, whereas the current survey and prior USA studies included all physiotherapists, irrespective of advanced training [28]. Accordingly, and as in the USA, physiotherapists possessing a musculoskeletal specialization were more likely to use SM and more comfortable when doing so. That is, it has been previously observed that SM is taught to a lesser extent within physiotherapy undergraduate programs and that advanced training has the potential to influence the reasoning, the decision-making, and the technical skills of professionals [33, 97, 98]. It is also important to note that a large amount of variation exists between the training and the content provided within programs and between countries [33]. Interestingly, using CPRs increases the use and the comfort of both Italian and USA physiotherapists, suggesting that using a decision-making tool that is supposed to help identify those patients that are most likely to favorably respond to the use of SM, decreases the level of concerns on SM safety.

Although no convincing evidence supports a causal link between cervical SM and SAEs [17, 18], and SM is still recommended in CPGs as an effective intervention to treat neck pain [8, 14, 15], similarly to the Netherlands and the UK [26, 27], we found a significant difference in the use of SM to the upper cervical spine when compared to other spinal regions. Also, respondents reported experiencing barriers to the use of upper cervical SM three times more than in the other spinal regions. Thus, physiotherapists worldwide seem to possess different beliefs about the perceived risks of upper cervical SM. The main causes reported in the literature appear related to anxiety on the safety of SM and the lack of clinical expertise in this spinal region. Notably, these barriers seem to be dogmatically influenced by traditional theoretical constructs (e.g., Maitland) [26]. The contrasting caution levels in the different spinal regions between SM and mobilization appear to be based on the empirical assumptions that upper cervical SM increases the risk of SAEs [40]. Notably, Michaeli reported SAEs more frequently following mobilization in different cervical regions [30]. Additionally, although Canadian physiotherapists commonly use less cervical SM because of a perceived association with SAEs [31], they reported having experienced minor to moderate adverse events with the same average occurrence for both mobilization and SM [21].

As well as physiotherapists from the Netherlands and the USA, Italian physiotherapists are generally comfortable performing SM [27, 28]; however, their comfort level drastically drops for the use of upper cervical SM. Interestingly, anxiety on safety as a barrier to perform SM was a major difference between Italian, UK, USA, and Dutch physiotherapists [26–28]. Our results showed that Italian physiotherapists were more focused on the patients’ beliefs and preferences instead of their anxiety surrounding safety, recognizing the patient-centered approach as a key feature of their practice [99]. Our results also demonstrated that working in a direct access setting and having more years of practice is associated with an increase in comfort level; that is, giving the opportunity to practice their skills increases the physiotherapists’ confidence [100–102]. In line with UK, USA, and the Netherlands, most respondents perceived “lack of practice/training” and “lack of clinical experience” as major barriers to performing SM for all spinal regions [26–28].

### Implications on clinical practice and future research

Although the general trend from contemporary systematic reviews suggests that SM is a valuable and cost-effective treatment for musculoskeletal pain [12, 103], Italian physiotherapists do not consider SM as an important core skill set. In addition to the anecdotal beliefs mentioned above, one primary reason may lie in the debate on the abandonment of hands-on interventions seen in, for example, social media-based opinions [12]. With the attempts to discredit SM, advocates find fertile ground to prove the reductionist juxtaposition between hands-on and hands-off approaches in the methodological flaws of SM primary studies. The results of the majority of SM clinical trials should be considered with caution, as they mainly rely on a traditional construct of SM—e.g., proper technique selection [7, 12], expecting one single popping sound from a single targeted joint [66–68, 75, 104], reliable/valid palpatory skills [105–107], correcting peripheral impairments [5, 7]–which has long since been outdated [5, 7]. Another confounding factor is tending to average results across heterogeneous substrates; more specifically, SM is skill-dependent in both the application and the execution, and there are technical differences between SM treatments done by osteopaths, chiropractors, and physiotherapists [1]. Nevertheless, the contention that SM is a real treatment with therapeutic effects and few harms, is even supported by the averaging of heterogeneous data [8, 108].

Although the traditional features of manual therapy have been strongly challenged [5–7], Italian physiotherapists still rely on biomechanical concepts to decide whether to use SM and to determine its effectiveness (e.g., evaluation of passive accessory inter-vertebral joint mobility). Importantly, SM has been found to involve both biomechanical and neurophysiological mechanisms [5]. However, although the interaction of these mechanisms has been frequently neglected, their combination could explain the reason for the effectiveness of SM despite its implementation heterogeneity. In addition, it might explain the irrelevance of SM traditional features on outcomes [5]. That is, continuing to attribute the effect of SM to peripheral biomechanical mechanisms is too simplistic. Therefore, SM should be re-conceptualized in a broader multidimensional framework that embraces the complexity of pain and respects the patient singularity [7, 105]. Such a comprehensive and dispositional approach accounts for the dynamic interplay between a myriad of factors, such as the sensory, cognitive, and affective processes, situationally influenced by expectations, mood, desires, culture, and past experiences [7]. Nonetheless, manual therapy, including SM, is a socio-culturally integral part of the professional identity that respects the patient’s expectation when they seek help from a physiotherapist [60]. Thus, SM remains an important skill within physiotherapy and does not merit being replaced because it might have been deemed to be outdated [109].

Many randomized controlled trials lack of pragmatism across the Rating of Included Trials on the Efficacy-Effectiveness Spectrum domains (i.e., they emphasize efficacy); additionally, randomized controlled trials are not designed to determine how moderators influence the treatment effect [110]. We believe that perhaps it is time to also focus on understanding how SM works and what are the mechanisms behind it. Future studies should attempt to establish links between the associated responses to SM and clinical outcomes, and the covariance of their changes. Therefore, there is a clear need for more focused research to understand what SM actually does, and how we might do it better; additionally, a mechanistic-based approach may provide a more robust approach to the design of clinical trials. Also, qualitative or mixed methods research should attempt to establish the nature of patients’ expectations and physiotherapists’ beliefs.

Our results suggest that anecdotal beliefs from prior educational background, research and practice are reciprocally influenced and lead to the propagation of misconception on the appropriate use of SM in clinical practice. However, specialization and updated programs seem to impact the implementation of current literature into practice. Given the paucity in the current literature, this article serves as an updated framework on the evidence-based use of SM. An infographic was designed for public use with the dual objective of raising awareness among physiotherapists about this subject and providing practical and easy to implement resources for the everyday use of SM in clinical practice (Additional file 3: Appendix 3).

### Strengths and limitations of this study

This study is built on existing surveys and adds scope to explore the differences with prior studies on this topic [27, 28]. One key strength is the rigorous survey’s developmental process in line with previous published surveys [27, 28, 48–50]. However, the content and face validity of the questionnaire could have been improved by statistical testing. Another limitation is that the survey was not translated forward and backward. The achievement of the required sample size confirms the willingness of physiotherapists to participate on this topic. Furthermore, although we did not send personal invitations, the publication of several reminders helped to reach a number of Italian physiotherapists in line with previous surveys [48–50]. Where the purpose of a study is to gain a general sense of a belief or attitude, a lower level of precision may be acceptable, and hence a smaller sample size may be drawn [47]. Although we obtained the required sample size calculation, this study does have limitations in the generalization of the results. Although the web-link to the survey was only distributed initially by email to the members of the Italian Physiotherapists Association, the participation invitations methods used in this survey are potentially subject to selection bias. As reminders were sent via mailing list and publicly posted on social media it is not possible to know how many people saw the reminders. Therefore, our results could be challenged in their generalizability. For example, this could potentially be an explanation for the relatively "young" sample in this study. Additionally, responder bias is possible because of the very detailed and specific questions in our survey, and the potential influence of the point of view of the survey respondents by the public debate of the topic within the profession should be considered. Although previous surveys endorse our results [27, 28], generalizability of findings may be limited, because we included only Italian physiotherapists. That is, educational standards between professions providing SM and national standards may differ.

## Conclusion

Our study observed that, although Italian physiotherapists did not consider it to be a core part of their skillset, SM is still regularly provided in contemporary clinical practice. Most physiotherapists who responded to our survey reported being comfortable in delivering SM and believe SM is safe and effective. Their comfort, safety perception and use significantly differed between the upper cervical spine and other spinal regions. These findings are aligned with previous studies conducted in SA, UK, Canada, USA, and the Netherlands. The odds of being more comfortable and perceiving SM to be safe were higher for those with more than 5 years of clinical experience and those aware of CPRs. In addition, male physiotherapists were more likely to be comfortable and to regularly provide SM in clinical practice. Our study is the first to report that a background in a traditional non-thrust manual therapy paradigm (i.e., Maitland) significantly influenced the physiotherapists’ attitude and beliefs about SM. Lack of formal clinical training and anxiety on safety have been observed as the main influencing factors. Anecdotal beliefs from prior educational background, research and practice are reciprocally influenced and lead to the propagation of misconceptions on the use of SM in clinical practice. To shed light on this topic, we proposed an updated framework on the evidence-based utilization of SM for clinical practice.

## Supplementary Information

**Additional file 1.** **Appendix** **1.** Survey questions.

**Additional file 2.** **Appendix** **2.** Post-*hoc* comparisons between response among the educational background.

**Additional file 3.** **Appendix** **3. **An updated framework on evidence-based Spinal thrust Manipulation.

## Data Availability

The datasets used and/or analyzed during the current study are available from the corresponding author on reasonable request.
