# Supplementary material for: Knowledge, beliefs, and attitudes of spinal manipulation: a cross-sectional survey of Italian physiotherapists
Source: Chiropr Man Therap. 2022 Sep 12;30:38. doi: 10.1186/s12998-022-00449-x (PMC9465888; doi:10.1186/s12998-022-00449-x)
Supplement: Supplementary file 1 — Additional file 1. Appendix 1. Survey questions. [file 12998_2022_449_MOESM1_ESM.docx]

Appendix 1. Survey questions. SM = high-velocity low-amplitude spinal thrust manipulation; OMPT = orthopaedic manipulative physical therapist; * = select ALL that apply.

**SECTION 1: DEMOGRAPHICS**

1. **What is your gender?**

Male

Female

1. **What is your age?**

*(Numeric continuous answer)*

1. **What is your highest earned degree?**

Bachelor

MSc

PhD

1. **Did you earn a Musculoskeletal (OMPT) specialization?**

No

Yes

1. **How many years have you been practicing as a licensed physiotherapist?**

0-5

6-10

11-15

16-20

20+

1. **What physiotherapy setting(s) do you currently practice in? ***

Private practice (primary care line)

Hospital (secondary care line)

Lecturer

Researcher

1. **What is your main access regimen?**

Direct access

Secondary care referral pathway

1. **Please estimate what percentage of your patients presenting with:**

Cervical complaints

Thoracic complaints

Lumbar complaints

Pelvic complaints

1. **Among your previous education, which one most influences your spinal manipulation practice?**

Continuing professional development course on SM

Musculoskeletal specialization

Physiotherapy undergraduate program

Traditional manual therapy program (e.g., Maitland)

Osteopathy post-graduate program

None

**SECTION 2: THE USE OF SPINAL MANIPULATION IN CLINICAL PRACTICE**

1. **Are you aware of any Clinical Prediction Rules for patients with neck, thoracic, or back pain who are more likely to benefit from spinal manipulation?**

Yes

No *(skip to question 13)*

1. **Which of these Clinical Prediction Rules are you familiar with? ***

Back pain that responds to lumbopelvic spine manipulation

Neck pain that responds to thoracic spine manipulation

Neck pain that responds to cervical spine manipulation

None

1. **Do you use the Clinical Prediction Rules to identify those patients who will benefit from spinal manipulation?**

Yes

No

***Please rate your level of agreement with the following statements:***

**13.1 SM is safe and effective for patients with upper cervical complaints**

Strongly agree

Agree

Neutral

Disagree

Strongly disagree

**13.2 SM is safe and effective for patients with cervical complaints**

Strongly agree

Agree

Neutral

Disagree

Strongly disagree

**13.3 SM is safe and effective for patients with thoracic complaints**

Strongly agree

Agree

Neutral

Disagree

Strongly disagree

**13.4 SM is safe and effective for patients with lumbar complaints**

Strongly agree

Agree

Neutral

Strongly Disagree

**14.1 Prior to a SM to the upper cervical spine, I usually perform an additional screening (e.g., pre-manipulative testing)**

Strongly agree

Agree

Neutral

Disagree

Strongly disagree

**14.2 Prior to a SM to the cervical spine, I usually perform an additional screening (e.g., pre-manipulative testing)**

Strongly agree

Agree

Neutral

Disagree

Strongly disagree

**14.3 Prior to a SM to the thoracic spine, I usually perform an additional screening**

Strongly agree

Agree

Neutral

Disagree

Strongly disagree

**14.4 Prior to a SM to the lumbar spine, I usually perform an additional screening**

Strongly agree

Agree

Neutral

Disagree

Strongly disagree

**15.1 I regularly perform SM to the upper cervical spine when patients require it.**

Strongly agree

Agree

Neutral

Disagree

Strongly disagree

**15.2 I regularly perform SM to the cervical spine when patients require it.**

Strongly agree

Agree

Neutral

Disagree

Strongly disagree

**15.3 I regularly perform SM to the thoracic spine when patients require it.**

Strongly agree

Agree

Neutral

Disagree

Strongly disagree

**15.4 I regularly perform SM to the lumbar spine when patients require it.**

Strongly agree

Agree

Neutral

Disagree

Strongly disagree

**16.1 I am comfortable performing SM to the upper cervical spine when patients require it.**

Strongly agree

Agree

Neutral

Disagree

Strongly disagree

**16.2 I am comfortable performing SM to the cervical spine when patients require it.**

Strongly agree

Agree

Neutral

Disagree

Strongly disagree

**16.3 I am comfortable performing SM to the thoracic spine when patients require it.**

Strongly agree

Agree

Neutral

Disagree

Strongly disagree

**16.4 I am comfortable performing SM to the lumbar spine when patients require it.**

Strongly agree

Agree

Neutral

Disagree

Strongly disagree

**17. Although rare, I explain potential adverse events to the patient before performing spinal manipulation.**

Always and in details

Always but summarily

Sometimes

Few times

Never

**SECTION 3: BARRIERS TO PERFORM SPINAL MANIPULATION**

**18.1 What do you feel a barrier to perform SM in the upper cervical spine: ***

Lack of education

Lack of practical training (manual skill)

Lack of experience

Concerns about its safety

Gaining informed consent

Lack of evidence for its effectiveness

Fear of the patients

None

**18.2 What do you feel a barrier to perform SM in the cervical spine: ***

Lack of education

Lack of practical training (manual skill)

Lack of experience

Concerns about its safety

Gaining informed consent

Lack of evidence for its effectiveness

Fear of the patients

None

**18.3 What do you feel a barrier to perform SM in the thoracic spine: ***

Lack of education

Lack of practical training (manual skill)

Lack of experience

Concerns about its safety

Gaining informed consent

Lack of evidence for its effectiveness

Fear of the patients

None

**18.4 What do you feel a barrier to perform SM in the lumbar spine: ***

Lack of education

Lack of practical training (manual skill)

Lack of experience

Concerns about its safety

Gaining informed consent

Lack of evidence for its effectiveness

Fear of the patients

None

**SECTION 4: KNOWLEDGE AND AWARENESS OF SPINAL MANIPULATION IN CLINICAL PRACTICE**

**19. What indicators mainly lead you to perform SM? ***

Pain

Range of Motion Reduction

Patient's request or expectation

Manual testing

Clinical Prediction Rules

**20. Which of the following are indicators for a successful technical delivery of SM? ***

Multiple popping sounds

Single popping sound

No popping sound

Dysfunction/positional-fault re-positioning/correction

Patient's symptoms improvement

Accessory/regional Range of Motion improvement

Perception of tension/stiffness reduction on the tissue

**21. What explanations do you provide to the patient regarding the popping sound? ***

Release/Re-positioning of a vertebral segment

Physical reactions of the intra-articular gas (inception/collapse)

Release/Re-positioning of a part vertebral disc/synovial or meniscoid fold

Tissue friction or relaxation

None

**22. Is the popping sound necessary for an effective SM?**

Yes

No

**23. During SM, Is the impulse specific to the target joint?**

Yes

No

**24. Is the popping sound specifically elicited from the target joint?**

Yes

No

**24. Which of the following manual therapy technique do you use as your first choice?**

Mobilization (without thrust)

Manipulation (with thrust)

**26. How important is SM in the physiotherapy's skillset?**

likert scale (0-100)
