# Supplementary material for: Knowledge, beliefs, and attitudes of spinal manipulation: a cross-sectional survey of Italian physiotherapists
Source: Chiropr Man Therap. 2022 Sep 12;30:38. doi: 10.1186/s12998-022-00449-x (PMC9465888; doi:10.1186/s12998-022-00449-x)
Supplement: Supplementary file 2 — Additional file 2. Appendix 2. Post-hoc comparisons between response among the educational background. [file 12998_2022_449_MOESM2_ESM.docx]

Appendix 2. Post-*hoc* comparisons between response among the educational background.

| **QUESTION** | **SPINAL REGION** | **MSK vs. CPD** | **MSK vs. OSTEO** | **MSK vs.PT** | **MSK vs. NONE** | **MSK vs. TRAD** | **CPD vs. OSTEO** | **CPD vs. PT** | **CPD, vs. NONE** | **CPD vs. TRAD** | **OSTEO vs. PT** | **OSTEO vs. NONE** | **OSTEO vs. TRAD** | **PT vs. NONE** | **PT vs. TRAD** | **NONE vs. TRAD** |
| --- | --- | --- | --- | --- | --- | --- | --- | --- | --- | --- | --- | --- | --- | --- | --- | --- |
| SM is safe and effective for patients with XXX spine complaints | Upper cervical (C0-3) | n.s. | n.s. | **<0.001** | **0.001** | **<0.001** | n.s. | **0.017** | **0.019** | **<0.001** | n.s. | n.s. | **0.001** | n.s. | n.s. | n.s. |
|  | Cervical (C3-7) | n.s. | **0.023** | **0.01** | **0.031** | **<0.001** | n.s. | n.s. | n.s. | **<0.001** | n.s. | n.s. | n.s. | n.s. | n.s. | n.s. |
|  | Thoracic | n.s. | **0.004** | n.s. | **0.004** | **<0.001** | n.s. | n.s. | n.s. | **0.002** | n.s. | n.s. | n.s. | n.s. | n.s. | n.s. |
|  | Lumbar | n.s. | n.s. | n.s. | n.s. | **<0.001** | n.s. | n.s. | n.s. | **<0.001** | n.s. | n.s. | **0.019** | n.s. | n.s. | n.s. |
| Prior to a SM to the XXX spine, I usually perform an additional screening | Upper cervical (C0-3) | n.s. | **<0.001** | n.s. | n.s. | **0.048** | **0.032** | n.s. | n.s. | n.s. | n.s. | n.s. | n.s. | n.s. | n.s. | n.s. |
|  | Cervical (C3-7) | n.s. | n.s. | n.s. | n.s. | n.s. | n.s. | n.s. | n.s. | n.s. | n.s. | n.s. | n.s. | n.s. | n.s. | n.s. |
|  | Thoracic | n.s. | n.s. | n.s. | n.s. | n.s. | n.s. | n.s. | n.s. | n.s. | n.s. | n.s. | n.s. | n.s. | n.s. | n.s. |
|  | Lumbar | n.s. | n.s. | n.s. | **0.002** | n.s. | n.s. | n.s. | **0.009** | n.s. | n.s. | n.s. | n.s. | n.s. | n.s. | n.s. |
| I regularly perform SM to the XXX spine when patients require it | Upper cervical (C0-3) | n.s. | n.s. | n.s. | 0.063 | **<0.001** | n.s. | n.s. | n.s. | **<0.001** | n.s. | **0.043** | **<0.001** | n.s. | n.s. | n.s. |
|  | Cervical (C3-7) | n.s. | n.s. | n.s. | **<0.001** | **<0.001** | n.s. | n.s. | **0.001** | **0.001** | n.s. | **0.004** | **0.012** | n.s. | n.s. | n.s. |
|  | Thoracic | n.s. | n.s. | **0.017** | **<0.001** | **<0.001** | n.s. | n.s. | **0.018** | **0.001** | n.s. | n.s. | **0.012** | n.s. | n.s. | n.s. |
|  | Lumbar | n.s. | n.s. | **0.023** | **<0.001** | **<0.001** | n.s. | n.s. | n.s. | **<0.001** | n.s. | n.s. | **0.013** | n.s. | n.s. | n.s. |
| I am comfortable performing SM to the XXX spine when patients require it | Upper cervical (C0-3) | n.s. | n.s. | **0.001** | **<0.001** | **<0.001** | n.s. | **0.003** | **0.001** | **<0.001** | **0.007** | **0.002** | **<0.001** | n.s. | n.s. | n.s. |
|  | Cervical (C3-7) | n.s. | n.s. | **<0.001** | **<0.001** | **<0.001** | n.s. | **0.006** | **<0.001** | **<0.001** | **0.021** | **0.001** | **<0.001** | n.s. | n.s. | n.s. |
|  | Thoracic | n.s. | n.s. | **0.002** | **<0.001** | **<0.001** | n.s. | **0.004** | **<0.001** | **<0.001** | n.s. | **<0.001** | **0.001** | n.s. | n.s. | n.s. |
|  | Lumbar | n.s. | n.s. | **0.002** | **<0.001** | **<0.001** | n.s. | **0.509** | **0.003** | **<0.001** | n.s. | **0.009** | **<0.001** | n.s. | n.s. | n.s. |

**Notes:** SM = high-velocity low-amplitude spinal thrust manipulation; MSK = musculoskeletal specialisation; PT = physiotherapy undergraduate program; CPD = continuing professional development courses on SM; OSTEO = Osteopathy post-graduate program; TRAD = traditional manual therapy post-graduate programs (i.e., Maitland); NONE = never been trained; n.s.= not significant. Significant p-values are reported in bold.
