# Supplementary material for: Knowledge, beliefs, and attitudes of spinal manipulation: a cross-sectional survey of Italian physiotherapists
Source: Chiropr Man Therap. 2022 Sep 12;30:38. doi: 10.1186/s12998-022-00449-x (PMC9465888; doi:10.1186/s12998-022-00449-x)
Supplement: Supplementary file 3 — Additional file 3. Appendix 3. An updated framework on evidence-based Spinal thrust Manipulation. [file 12998_2022_449_MOESM3_ESM.pdf]

AN UPDATED FRAMEWORK ON EVIDENCE-BASED SPINAL THRUST MANIPULATION

Appendix 3.

Our study confirms that the beliefs of physiotherapists worldwide on the safety and effectiveness, comfort in delivery, and clinical use of high-velocity low-amplitude spinal thrust manipulation (SM) significantly differs between the upper cervical spine and other spinal regions. Prior educational background (especially traditional manual therapy approaches), lack of formal training in SM, and anxiety regarding the safety of upper cervical SM have been observed as the main influencing factors. Anecdotal beliefs from prior educational background, research and practice are reciprocally influenced and lead to the propagation of misconceptions on SM. The following infographic provides an updated framework on evidence-based SM.

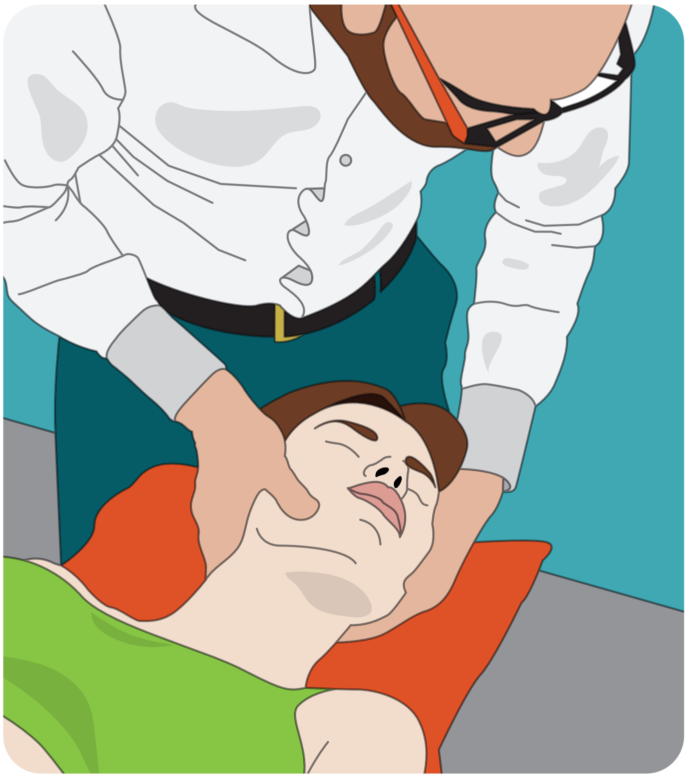

MYTHS	VS FACTS
**FEATURE**	
Many traditional manual therapy approaches associated with the use of spinal manipulation no longer inform the clinical application of SM.	The traditional manual therapy paradigms still influence physioterapy practice, research, and education: there is a need for a standardization in the framework surrounding and the clinical application of sm by physiotherapists.
**DEFINITION**	
SM is a progression of mobilization (theoretical construct based on force/risk exposure).	SM and mobilization are different interventions and therefore require different psychomotor skills for the successful delivery.
End-range manoeuver combining multiple physiological levers (that enters the para-physiological range).	SM is likely a mid-range manoeuvre combining multiple accessory and physiological levers.
One single pop is the most specific for the target level.	Multiple popping sounds should be expected with the use of SM in any spinal region.
The popping sound is not necessary to define a successful delivery of SM.	The presence of the popping sound is one criterion to define a successful delivery of SM (for both the patient and practitioner).

MYTHS	FACTS
**APPLICATION**	
Vertebral Subluxation complex;<br>Positional faults;<br>Coupling theory (e.g., Fryette's laws);<br>Convex-Concave Rule;<br>Cyriax's capsular pattern;<br>End-feel classification;<br>Disc displacement with positions;<br>Reliable/valid palpatory skills and "feeling" of passive accessory or physiological intervertebral motion testing;<br>Postural assymmetry and impairments;<br>Limb length discrepancy;<br>Organ dysfunction.	Rather than what the physiotherapist palpates, the location of the pain reported by the patient is the most useful item for identifyfing which vertebral segments should targeted with the SM.
**EXECUTION/SKILL**	
Directional specificity is required when selecting the appropriate SM technique to address the corresponding positional fault or movement restriction.<br>The production of a single popping suggests greater target specificty has been achieved with the SM.	Directional specificity is not required for the successful application of SM techniques for the reduction of pain and disablity.
Precise implementation of the thrust in single plane of motion.	The final thrust is often a multidirectional impulse.
Force to overcome the elastic barrier and enter the paraphysiological space (theoretical construct).	The main feature of SM is velocity | thrust duration related to loading rate/rate of force application.
**SAFETY**	
Mobilization is safer than SM, especially in the cervcial spine.	The risk of adverse events is no different for mobilization compared with manipulation for all spinal regions including the upper cervical spine.<br>Currently, there is no evidence that SM endanger vital structures, such as the spinal cord and the vertebral arteries.
Clinical prediction rules are necessary for subgrouping candidates.<br>Pre-manipulative testing is valid and reliable for identifying instabilility and vertebrobasilar insufficiency.	Clinical prediction rules that claim to identify those patients that will likely benefit from SM have been found to be not valid.<br>Premanipulative functional testing prior to the use of cervical SM should be abandoned because of the sensitivity, specificity and reliability of these tests are either unknown or too low for clinical use.
**RATIONAL**	
Biomechanical model/Correcting peripheral impairment in the local tissues.	SM should be re-conceptualized in a broader multidimensional framework.
**PHILOSOPHY**	
Abandon hands-on interventions.	Like all hands-on interventions, SM remains an important part of the physiotherapy skill-set and respect the patient's expectation and clinician's expertise.
